# PDGF receptor alpha inhibition induces apoptosis in glioblastoma cancer stem cells refractory to anti-Notch and anti-EGFR treatment

**DOI:** 10.1186/1476-4598-13-247

**Published:** 2014-11-08

**Authors:** Carlo Cenciarelli, Hany ES Marei, Manuela Zonfrillo, Pasquale Pierimarchi, Emanuela Paldino, Patrizia Casalbore, Armando Felsani, Angelo Luigi Vescovi, Giulio Maira, Annunziato Mangiola

**Affiliations:** Institute of Translational Pharmacology-CNR, Roma, Italy; Department of Cytology and Histology, Mansoura University, Mansoura, Egypt; Institute of Cell Biology and Neurobiology, Roma, Italy; Department of Biotechnologies and Biosciences, University of Milan-Bicocca, Milan, Italy; Institute of Neurosurgery, Catholic University-School of Medicine, Roma, Italy

## Abstract

**Background:**

Cancer stem cells (CSC) represent a rare fraction of cancer cells characterized by resistance to chemotherapy and radiation, therefore nowadays there is great need to develop new targeted therapies for brain tumors and our study aim to target pivotal transmembrane receptors such as Notch, EGFR and PDGFR, which are already under investigation in clinical trials setting for the treatment of Glioblastoma Multiforme (GBM).

**Methods:**

MTS assay was performed to evaluate cells response to pharmacological treatments. Quantitative RT-PCR and Western blots were performed to state the expression of Notch1, EGFR and PDGFRα/β and the biological effects exerted by either single or combined targeted therapy in GBM CSC. GBM CSC invasive ability was tested in vitro in absence or presence of Notch and/or EGFR signaling inhibitors.

**Results:**

In this study, we investigated gene expression and function of Notch1, EGFR and PDGFR to determine their role among GBM tumor core- (c-CSC) *vs*. peritumor tissue-derived cancer stem cells (p-CSC) of six cases of GBM. Notch inhibition significantly impaired cell growth of c-CSC compared to p-CSC pools, with no effects observed in cell cycle distribution, apoptosis and cell invasion assays. Instead, anti-EGFR therapy induced cell cycle arrest, sometimes associated with apoptosis and reduction of cell invasiveness in GBM CSC. In two cases, c-CSC pools were more sensitive to simultaneous anti-Notch and anti-EGFR treatment than either therapy alone compared to p-CSC, which were mostly resistant to treatment. We reported the overexpression of PDGFRα and its up-regulation following anti-EGFR therapy in GBM p-CSC compared to c-CSC. RNA interference of PDGFRα significantly reduced cell proliferation rate of p-CSC, while its pharmacological inhibition with Crenolanib impaired survival of both CSC pools, whose effects in combination with EGFR inhibition were maximized.

**Conclusions:**

We have used different drugs combination to identify the more effective therapeutic targets for GBM CSC, particularly against GBM peritumor tissue-derived CSC, which are mostly resistant to treatments. Overall, our results provide the rationale for simultaneous targeting of EGFR and PDGFR, which would be beneficial in the treatment of GBM.

**Electronic supplementary material:**

The online version of this article (doi:10.1186/1476-4598-13-247) contains supplementary material, which is available to authorized users.

## Introduction

Glioblastoma multiforme (GBM) is an aggressive type of brain cancer that resists treatment. Recently, it has been established that in many types of cancer the bulk of cells that make up a tumor are derived from a small population of CSC, also known as tumor initiating cells. CSC are distinguished from the bulk of the population of tumor cells by their ability to successfully seed new tumors when implanted in low numbers into experimental animals. Such cells are proposed to persist in recurrent GBM and to have enhanced resistance to chemotherapy and radiation-induced apoptosis [1].

The Notch pathway plays an important role in cellular processes during embryonic and postnatal development, including stem cells renewal, cell fate determination and apoptosis. A role for Notch signaling in the maintenance of cancer stem cells has been described in preclinical models and recently in clinical studies [2–4]. Increasing evidence demonstrates a deregulated expression of Notch receptors and Notch ligands in GBM, and their knockout inhibit gliomas proliferation and survival. Therefore this pathway could be considered as a therapeutic target for cancer therapy [5, 6]. Notch receptor is synthesized as an inactive 300 kDa precursor protein, which is proteolytically cleaved by a furin-like convertase before it inserts as a non-covalently bound heterodimer in the plasma membrane There are four Notch receptors (Notch1, 2, 3 and 4) and five ligands of the DSL (Delta and Serrate Ligands) family, which includes Delta-like ligands (DLL) 1, 2, 3, and Serrate/Jagged (JAG) 1 and 2. Members of both the Notch receptor and DSL ligand families are, for the most part, type I single-pass integral membrane proteins. Signaling is initiated by binding of a Notch ligand expressed on one cell to a Notch receptor on an adjacent cell. Upon ligand binding to the receptor, Notch is sequentially cleaved by ADAM10/TACE (a disintegrin and metalloproteinase) and by a presenilin-dependent γ-secretase protease complex which consists of presenilin 1 (PSEN1) or PSEN2, Nicastrin, PEN2 and APH1 [7]. This process results in the release in the cytoplasm of a soluble fragment consisting of the entire intracellular domain, termed Notch intracellular domain (NICD). After translocation to the nucleus, NICD binds to transcription factor CSL, and converts a large co-repressor in an active transcriptional complex that activates the transcription of Notch target genes. These include genes encoding Hairy Enhancer of Split (HES1), HES-related proteins (HEY), p21^waf1^, Cyclin D1 and 3, c-myc, and HER2 [8–11].

A number of genetic alterations are responsible for the malignancy of GBM, often mutations leading to the hyperactivation of receptor tyrosine kinases (RTK). A combination of proteomic and genomic analyses of 243 GBM from The Cancer Genome Atlas (TCGA), subdivides these tumors into three subclasses based on the pattern of expression and genetic alterations: classical/EGFR+, proneural/PDGFR + and mesenchymal/NF1+ classes [12]. Le Mercier et al. [13], report that the addition of temozolomide to conventional radiotherapy significantly improved the survival of patients belonging to the classical subtype, but it did not affect the survival of patients belonging to the proneural subtype, suggesting the importance to clinically subdividing patients in order to devise the best targeted therapy. EGFR is overexpressed in almost 40-50% of GBM and contributes to uncontrolled proliferation and survival of glioma cells [14]. A well-documented alteration in GBM is the amplification and activating mutation of EGFR variant III (EGFRvIII). EGFRvIII has an overall prevalence of almost 60% in patients whose tumors show amplification of wild type (wt) EGFR [15, 16]. Enhanced activation of RTK leads to the activation of intracellular signaling pathways such as the Raf/MEK/Erk and the PI3K/Akt pathways, which are ultimately responsible for the malignant phenotype of glioma cells [17].

Another subset of gliomas, the PDGFR subclass account for 25-30% of GBM, and is characterized by dysregulation of PDGFR activity, which in some cases is due to amplification and rearrangements of the PDGFRα gene locus, and in others to overexpression of the PDGF ligands [12, 18, 19]. PDGFR is a transmembrane receptor with 5 immunoglobulin-like repeats in its extracellular domain and a tyrosine kinase in its intracellular domain. There are four PDGF ligands (PDGF-A, PDGF-B, PDGF-C and PDGF-D) that dimerize and bind to PDGF receptors, PDGFRα and PDGFRβ [20]. PDGF-A, PDGF-B, and PDGF-C bind to PDGFRα; PDGF-B and PDGF-D bind to PDGFRβ. The binding of a ligand to the receptor induces its autophosphorylation and activation of pivotal intracellular signals (MAP kinase, PI3K/Akt, JAK/STAT and PLC-PKC), which results in proliferation, survival, migration and oncogenesis [21]. Recently, Kim et al. [22] report that genetic or pharmacological targeting of PDGFRβ in selected CD133 positive GBM CSC (but not PDGFRα), attenuated self-renewal, survival, tumor growth and invasion. Therefore, development of specific therapies using tyrosine kinase inhibitors (TKI) targeted toward CSC holds promise, but cancer cells may acquire resistance to TKI. In fact, it has been reported that EGFR-mutant glioblastomas may evade EGFR TKI by transcriptionally de-repressing PDGFRβ [23].

The current study aims to devise strategies to selectively target pivotal plasma membrane receptors relevant to maintenance of either core tumor- (c-CSC) or peritumor tissue (p-CSC)-derived GBM cancer stem cells. We investigated the biological effects on these classes of CSC mediated by TKI targeting individually Notch1, EGFR and PDGFR signaling respectively. Combination therapy with EGFR and Notch inhibitors provided more significant therapeutic effects than single therapy alone, promoting in some cases more apoptosis in c-CSC than p-CSC. The combination of EGFR and PDGFR inhibitors sensitizes either the most resistant GBM p-CSC or c-CSC to apoptosis. This finding is highly relevant because the peripheral area of the tumor is the site of recurrent GBM in 90% of cases. The *in vitro* pharmacological studies on CSC are a such compelling model as they hold the potential to develop new therapeutic strategies before employing them in clinical trials.

## Results

### GBM CSC culture and evaluation of Notch1 and RTKs gene expression

Cancer stem cells from GBM were isolated using defined criteria set up by neurosurgeons as described previously [24, 25]. We can summarize these briefly: lesion removal was achieved with resection margins that included the tumor and the neighboring, apparently normal tissue (between 1-2 cm from the tumor border; larger resections were performed in tumors that grew far from eloquent areas), which were removed entirely en bloc. Neuronavigation and intraoperative ultrasound were used to maximize the extent of intracranial tumor resection. From this bulk we retrieved either core- (c-CSC) or peritumor tissue-derived cancer stem cells (p-CSC). Cytogenetic and molecular analysis showed that the two types of CSC have quite diverse tumorigenic potential and distinct genetic anomalies [24]. Neurospheres of different sizes were obtained from cores of multiple specimen of GBM patients; these continued to propagate in suspension in long-term culture. CSC derived from peritumor tissue of GBM at early passages exhibited a different phenotypic behavior compared to c-CSC: they grew at a slow rate, forming small spheres, most of them attached to the plastic dishes. These latter particular morphological features, in some cases, were gradually lost at late passages in culture (data not shown).

**Figure 1:**
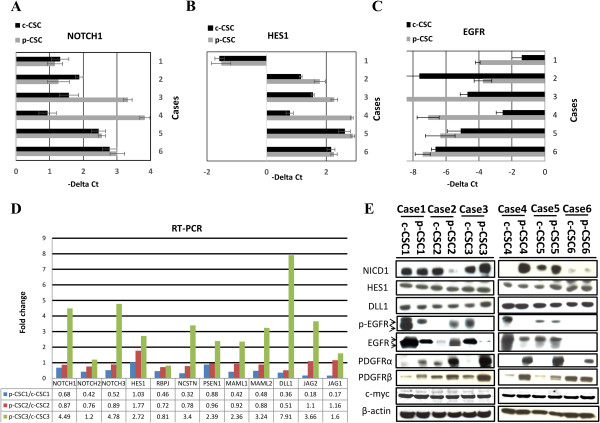
**RT-PCR and protein expression analysis in GBM core- and p-CSC. (A-B)** NOTCH1 and his gene target HES1 mRNA levels are highly expressed in p-CSC3 and p-CSC4 compared to relative counterparts c-CSC. **C**, EGFR mRNA expression results more abundant in c-CSC of cases 1-3-4, but in case 2 occurs the opposite. **(D)** Custom RNA macroarray analysis performed in the first three cases reveals the up regulation of several components of Notch pathway in p-CSC3, while the opposite was detected in Cases 1 and 2. **(E)** Western blot analysis of more relevant RTK in GBM: EGFR (p-EGFR), PDGFRα and β isoforms and the active Notch1 (NICD1), Notch ligand delta-like 1 (Dll1) and Notch1 gene target Hes1. PDGFRα abundance is an hallmark property of all p-CSC pools which suggests the presence of a cell subpopulation in p-CSC with probably distinct growth factor response. c-Myc is abundantly expressed in all pairs of CSC examined. Arrows denote the doublet of EGFR full-length and variant III. Error bars represent the mean ± SD (n = 3).

To understand how Notch1 and epidermal growth factor receptor (EGFR) signaling would affect cell growth and survival of GBM CSC, we first assessed the mRNA expression profile in six human cases, consisting of paired samples of c-CSC and p-CSC, for a total number of twelve CSC. RT-PCR experiments for NOTCH1, HES1, EGFR wt and variant EGFRvIII, were performed in triplicate for each sample and the relative expression reported as -∆Ct (Figure 1A-C). Notably, the p-CSC3 and p-CSC4 showed a significant up regulation of NOTCH1 gene compared to relative c-CSC, either at mRNAs level or the protein content of the Notch intracellular domain 1 NICD1, (the active form of Notch1) (Figure 1A, E). We carried out in parallel a custom RT-PCR array in the most studied cases (cases 1-3), which revealed and confirmed the up modulation of Notch signaling components in p-CSC3 versus c-CSC3, expressed as fold change (Fc) and including: NOTCH3 (4.78 Fc), Nicastrin (NCSTN, 3.4 Fc), Presenilin1 (PSEN1, 2.39 FC), Mastermind-like 1-2 (MAML1, MAML2, 2.36 and 3.24 FC respectively), Delta-Like Ligand 1, (DLL1, 7.91 Fc), and Serrate Ligand Jagged2, (JAG2, 3.66 Fc) (Figure 1B, C). The high mRNA levels of HES1, a Notch1 primary target gene, directly correlated to those of Notch1 in p-CSC3 and p-CSC4, suggesting a Notch1 dependent mechanism for Hes1 gene regulation (Figure 1A, B). Conversely, the high levels of HES1 mRNA inversely correlated to Notch1 gene expression in p-CSC2 (Figure 1A, B), suggesting that other signals converged in case-2 for HES1 gene transcription. A custom RT-PCR array for genes encoding Notch signaling components confirmed the reduction of Notch1 activation in p-CSC2 as well as NICD1 protein expression as compared to c-CSC2 (Figure 1D, E). Hes1 protein was detected in all CSC, raising the possibility that further mechanisms may contribute to Hes1 protein stability through the sonic hedgehog pathway as well as post-translational processes [26, 27].

The only mRNA positivity relative to EGFRvIII was found in c-CSC1, which represent around 16% of the specimens studied. EGFR mRNA expression was up modulated in c-CSC1, 3 and 4, down modulated in c-CSC2 compared to relative counterparts, or unmodulated in the rest of the cases (Figure 1C). EGFR protein expression mirrored the levels of transcripts in the same samples. We extended our protein analysis to another RTK, the platelet derived growth factor receptor isoforms α and β (PDGFRα/β). The inherent over expression of PDGFRα in p-CSC pools suggests this as a molecular signature of the GBM peritumor tissues, while PDGFRβ was expressed either in c-CSC or p-CSC of the six cases examined (although we have documented increasingly expression levels in p-CSC pools except for Case 6) (Figure 1E). We also reported the over expression of c-Myc, a direct target of Notch1 [10], which was equally express in all CSC.

### Differential response of GBM CSC to treatment with GSI-X and AG1478

**Figure 2:**
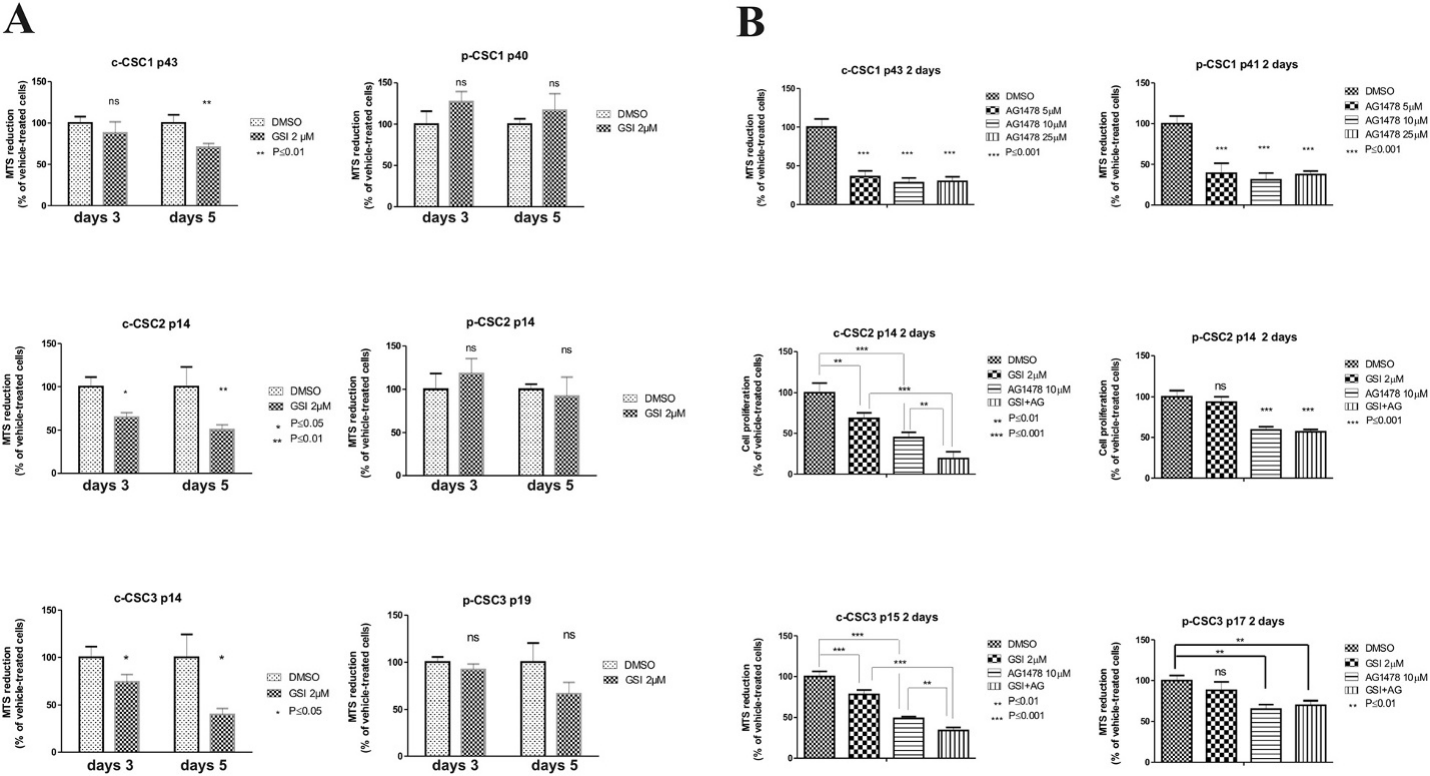
**Differential response of the GBM CSC to GSI-X and AG1478 treatment. (A)** GSI-X negatively affect cell proliferation of GBM c-CSC pools after 3 and 5 days of treatment as compared to p-CSC pools by MTS assay. **(B)** GBM CSC proliferation decrease after 2 days by single EGFR inhibition. Concurrent EGFR and Notch1 inhibition results in a synergistic anti-proliferative effect in c-CSC2 and c-CSC3 pools as compared to relative p-CSC pools. Error bars represent the mean ± SD (n = 3). All data shown are representative of results obtained from experiments conducted three times.

**Figure 3:**
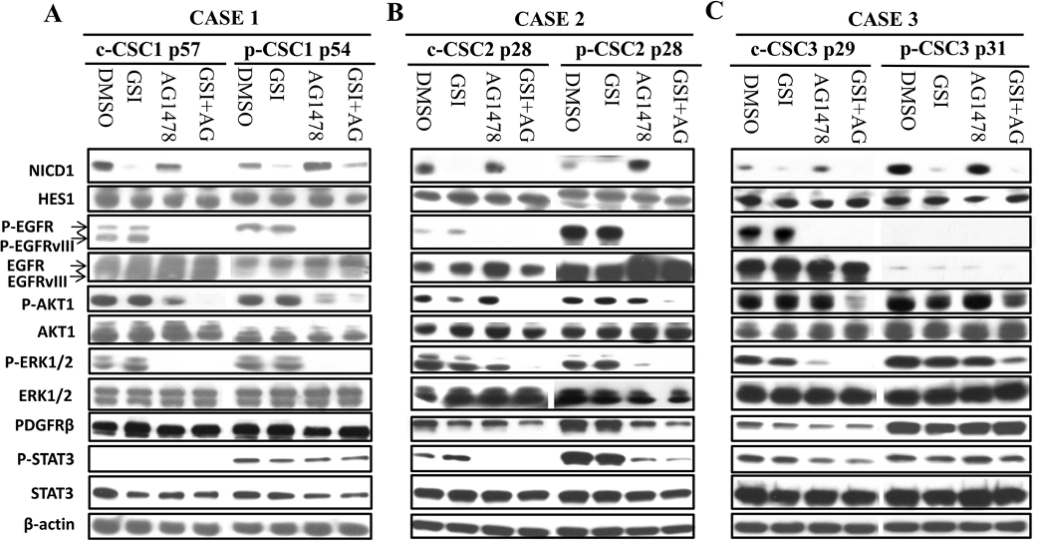
**Dual inhibition contributes to down modulation of PI3K/Akt, ERK1/2 and Jak/STAT3 pathways in GBM CSC. (A)** Western blots in Case 1 reveal none modulation of Notch1 target Hes1 protein by GSI-X. EGFR inhibition affects the phosphorylation status either of the full-lenght or EGFRvIII. p-Akt1 (S473) and p-ERK1/2 (T202/Y204) disappear with the dual treatment in both of pools, but p-Stat3 (Y705) and PDGFRβ expression was maintained high in p-CSC1 after treatment, and probably contribute to drug resistance. **(B)** Western blot analysis reveal no modulation of Notch1 target Hes1 protein in both pools of Case 2. Decrease of p-Akt1 protein levels by GSI-X in c-CSC2 but not in p-CSC2. Either p-Erk1/2 or p-Akt1 disappear following dual treatment. Higher levels of p-Stat3 in p-CSC2 compared to c-CSC2 may contribute to drug resistance as observed in p-CSC1. AG1478 alone or combined with GSI-X downmodulates the levels of p-Stat3 and PDGFRβ. **(C)** Western blot analysis detects significant differences in NICD1, EGFR and PDGFRβ protein expression between core- and p-CSC3. c-CSC3 show no modulation of p-Akt1 and p-Erk1/2 by Notch inhibition, while AG1478 causes p-Erk1/2 decrease but not p-Akt1. Dual treatment triggers a decrease of p-Stat3 with a significant loss of p-Akt1 in c-CSC3. On the contrary, p-CSC3 pool maintains an high expression of p-Akt1, p-Erk1/2, PDGFRβ and p-Stat3 either after AG1478 or dual treatment, which may explain the high cell drug resistance. Arrows denote the doublet of EGFR full-length and EGFRvIII.

CSC undergoing treatment with γ-secretase inhibitor-X (GSI-X), a Notch signaling inhibitor, either alone or along with an EGFR signaling inhibitor AG1478, were evaluated for any changes in cellular response by MTS assay. Here, the experiments were conducted only on cases 1, 2 and 3 (Figure 2). c-CSC2 and c-CSC3 were significantly sensitized with 2 μM of GSI-X after 3 days. Conversely, p-CSC2 showed an increase of cell proliferation, while p-CSC3 were not affected even at higher concentration of GSI-X (Additional file 1: Figure S1). In Case 1, both c-CSC1 and p-CSC1 showed resistance to the treatment after 3 days (Figure 3A); instead the treatment on the 5th day dropped proliferation of c-CSC1 to 70 ± 4.6% (Figure 2A). The treatment with 10 μM of AG1478 resulted in a significant sensitization of all six CSC as detected by MTS assay (Figure 2B).

No close correlation between sensitization to AG1478 with the levels or the mutational state of EGFR gene was found (Figure 1C, E). In fact, cell proliferation decreased to 28.3 ± 6.1% and 30,9 ± 8.3% in c-CSC1 and p-CSC1, respectively (Figure 2B), although c-CSC1 would overexpress both full-length and the variant III of EGFR (Figure 1E). In c-CSC2 and c-CSC3 cell proliferation dropped to 44,8 ± 6.4% and 49 ± 2.1% respectively against to 59.2 ± 4.1% of p-CSC2 and 65.2 ± 5.5% of p-CSC3. The treatment with the two inhibitors caused a synergistic anti-proliferative effect in c-CSC2 and c-CSC3, bringing cell proliferation down to 19 ± 8.3% and 34 ± 3.8% respectively (Figure 2B), while growth of p-CSC2 and p-CSC3 remained unmodified at 57 ± 2.9% and 70 ± 5.6%, respectively. This finding argued for a stronger anti-Notch1 and EGFR resistance of p-CSC *vs*. c-CSC pools, at least in these 2 cases.

To substantiate the effects observed by MTS assay, we carried out Western blots analysis for the main signal molecules relevant for proliferation and cell survival (Figure 3). The concentration of the inhibitors used in our treatment was effective in inhibiting Notch1 cleavage and EGFR phosphorylation on Try1068. Notably, only c-CSC1 showed a protein doublet of 180 and 140 KDa (corresponding to the full-length and EGFRvIII respectively); the rest of cases displayed the full lenght isoform (Figure 3). We evaluated the activation and modulation of the main downstream effectors of EGFR signaling, such as Akt1, Erk1/2 and Stat3, after two days of treatment. No significant changes of phosphorylation state of p-Akt1 and p-Erk1/2 were found in the three cases examined after 2 days of treatment with GSI-X. p-CSC3 displayed resistance even to higher concentration of GSI-X (Additional file 1: Figure S1). Conversely, p-Erk1/2 was suppressed in case 1, and significantly down modulated in case 2 and 3, except for p-CSC3, following AG1478 treatment. The combination of the two agents suppressed p-Erk1/2 expression in case 2 and 3, but it was still preserved in p-CSC3 and similarly results were seen for p-Akt-1 activation. Stat3 phosphorylation on Y705 has been shown to be directly mediated by EGFR or indirectly by proteins of JAK family. EGFR signaling inhibition caused a decrease of phosphorylation of p-Stat3 in case 2 and c-CSC3, conversely in p-CSC1 and p-CSC3 the levels of p-Stat3 were unmodulated, suggesting a mechanism of resistance to EGFR TKI treatment in these cases. PDGFRβ was more expressed in p-CSC than in c-CSC pools and unmodulated by either GSI-X or AG1478 alone, except for CSC2 that showed PDGFRβ down modulation by AG1478 treatment (Figure 3A-C).

### Simultaneous GSI-X and AG1478 treatment trigger apoptosis in core-CSC

**Figure 4:**
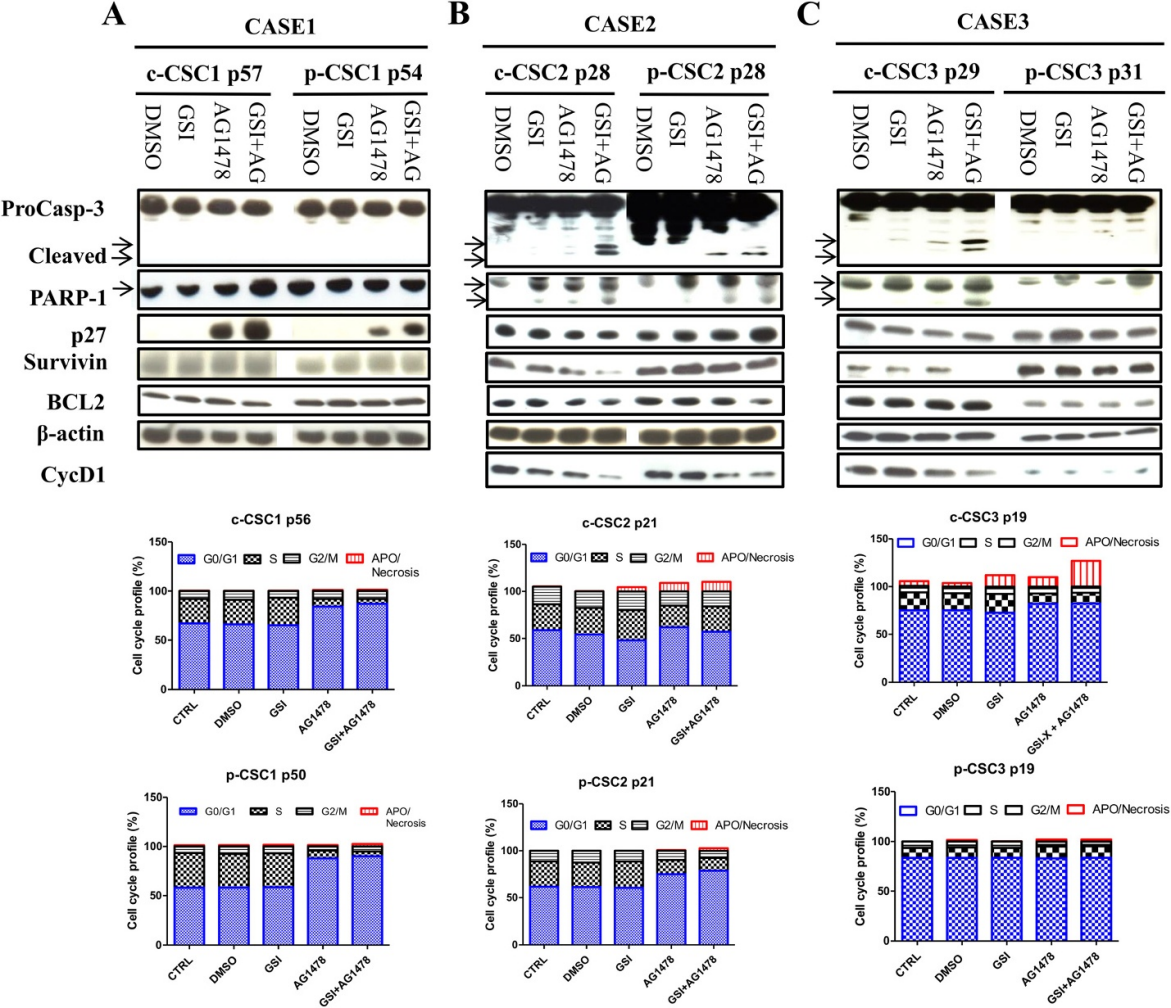
**Simultaneous targeting of Notch1 and EGFR signaling induces apoptosis in GBM c-CSC2 and c-CSC3 but not in CSC1. (A)** No induction of Caspase-3 and PARP-1 fragments cleavage following drug exposure was monitored. AG1478 induces an high expression of cell cycle regulatory protein p27^kip^ in c-CSC1 and p-CSC1. This finding correlates to a strong shifting from S to G_0_/G_1_ phase of cell cycle in both pools as observed by Flow cytometry. CycD1 was not detected by immunoblotting. **(B)** Appearance of Caspase-3 (17-19 KDa fragments) and PARP-1 (116-89 KDa) fragments are monitored in c-CSC2 either in presence of AG1478 or in combination with GSI-X, while in the p-CSC2 this phenomenon is present but at lesser extent. GSI-X treatment induced a modest apoptosis/necrosis effect preferentially in c-CSC2 and did not affect cell cycle distribution. EGFR signaling inhibition induces apoptosis/necrosis only in c-CSC2 pool, while AG1478 induces a consistent shifting to G_1_/G_0_ in p-CSC2 respect to c-CSC2, which correlated with higher levels of p27^kip^. Cyclin D1 protein is down modulated in both cell populations of case 2 by EGFR inhibition, instead the antiapoptotic protein Survivin declines preferentially in c-CSC2 by the dual blockade, suggesting that the combination therapy is more effective in the c-CSC2 than in p-CSC2. **(C)** GSI-X and AG1478 concurrently added in c-CSC3 resulted in Caspase-3 and PARP-1 fragments cleavage, decrease of both Cyclin D1 and Survivin expression. AG1478 induces a shift to G_0_/G_1_ phase in c-CSC3 and the drugs combination potentiates the apoptotic effect in c-CSC3. p-CSC3 are refractory to any drug blockade as reported by flow cytometry and Western blots analysis. Lower arrows indicate the fragments cleavage of Caspase-3 and PARP-1.

The main biological responses of cancer stem cells described in the literature following treatment with Notch inhibitors are: arrested cells and induction of apoptosis. Flow cytometry analyses showed a moderate apoptosis/necrosis induced by GSI-X, which resulted 4.7% and 12% in c-CSC2 and c-CSC3 respectively respect to 0.3% and 5% in DMSO-treated cells (Figure 4B, C). Single AG1478 treatment was more effective than GSI-X, as c-CSC2 and c-CSC3 were both shifted in G1 phase (61.8% and 82.1% respectively as compared to 54.2% and 74.5% in DMSO-treated cells) and induced to apoptosis/necrosis (9% and 10%, respectively).

Dual treatment raised apoptosis/necrosis to 10.2% in c-CSC2 and 27% in c-CSC3. These results were verified by induction of Caspase-3 and PARP-1 cleavage, reduction of both Survivin and Cyclin D1 as reported by Western blots (Figure 4B, C). AG1478 determined the accumulation of p-CSC2 in G1 phase (74,9%), while the dual treatment raised this to 78,7% compared to 61.3% in DMSO-treated cells. The slowing of cell cycle progression was associated with loss of Cyclin D1 and increase of p27^Kip^, but we monitored very low levels of Caspase-3 and PARP-1 cleavage fragments, and no effect on Survivin protein levels. Conversely, p-CSC3 were fully resistant to treatment (Figure 4C). EGFR inhibition induced cell cycle arrest of both c-CSC1 and p-CSC1 which amounted to 84.3% and 87.9% respectively versus 66.2% and 58.1% in DMSO-treated cells. This finding correlated with high induction of p27^Kip1^ (Figure 4A). Cyclin D1 was not detected by Western blots, although mRNAs were monitored by custom RT-PCR array (data not shown).

### Cell invasion properties of GBM CSC

**Figure 5:**
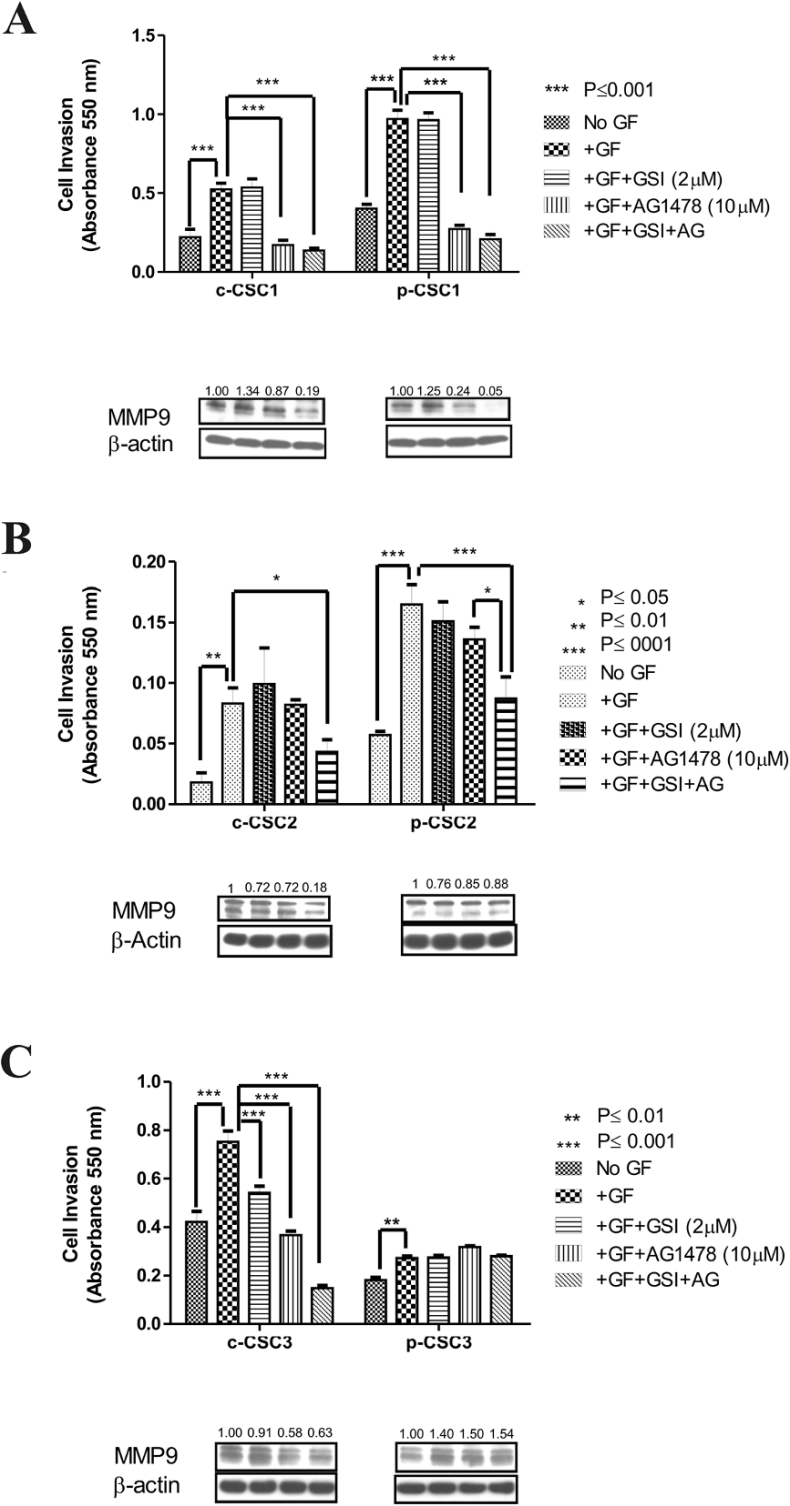
**Cell invasion properties of GBM CSC. (A)** In absence of GF p-CSC1 have higher invasive ability than c-CSC1 on matrigel invasion assay. Addition of GF promotes invasiveness of both cell populations of case 1, which is blocked by AG1478. Drugs combination don’t improve the effect of monotherapy but induces a strong repression of MMP9 protein expression. The values of MMP9 repression are calculated respect to DMSO-vehicle. **(B)** In absence of GF, p-CSC2 have higher invasive ability than c-CSC2. Addition of GF promotes the invasiveness of both cell populations of case 2 but only the combination of GSI-X and AG1478 significantly impairs the invasive performance of c-CSC2 and p-CSC2, which correlates to reduction of MMP9 protein. The values of MMP9 repression are calculated respect to DMSO-vehicle. **(C)** In absence of GF c-CSC3 holds higher invasive ability than p-CSC3 and similarly to other cases the addition of GF favors significantly their invasive features. AG1478 impairs c-CSC3 invasion but not p-CSC3. Moreover, drug combination increases the effect of AG1478 monotherapy only in c-CSC3. This finding correlates to a decline of MMP9 protein expression. Conversely, p-CSC3 shows drug resistance and retains high levels of MMP9. The values of MMP9 repression are calculated respect to DMSO-vehicle. Error bars represent the mean ± SD (n = 3). All data shown are representative of results obtained from experiments conducted three times.

In order to assess the invasion capabilities of CSC and the effects of the inhibitors either alone or in combination a matrigel invasion assay was performed. To invade the cells need an active process that involves cell motility and extracellular matrix proteolysis, and for this reason we monitored the expression of MMP-9, matrix metallopeptidase-9, which belongs to MMP family involved in tumor progression, including invasion and cancer metastasis [28]. Cell invasion ability was assessed in cases 1, 2 and 3 in absence or presence of growth factors (GF). In absence of GF, both p-CSC1 and p-CSC2 hold higher invasive ability compared to relative counterparts; conversely, c-CSC3 are much more invasive than p-CSC3. Following addition of GF, all CSC moved significantly through the plastic insert of the trans-wells dishes (Figure 5A-C). GSI-X treatment did not affect the invasive ability of any CSC, except for c-CSC3, although other authors report that genetic Notch1 knockdown resulted in reduced cell migration invasion in a glioma cell line by inhibition of β-catenin and NF-κB signaling [29].

It has been reported that EGFR inhibition efficiently blocked EGF-induced activation of MMP9 and cancer invasiveness [30]. In our work AG1478 treatment induced differential effects, in part explained by the RTK profile. AG1478 inhibited cell invasion and MMP9 protein expression in c-CSC1 and p-CSC1, whereas the combination therapy resulted in suppression of MMP-9 (Figure 5A). AG1478 alone did not affect the invasive ability of c-CSC2 and p-CSC2, but the dual treatment influenced significantly both pools of cells and directly correlated with a stronger reduction of MMP-9 in c-CSC2 than in p-CSC2 (Figure 5B). Similar outcomes were obtained in c-CSC3, and the influence of this combination treatment was superior to monotherapy with AG1478. Conversely, p-CSC3 had a modest invasion ability compared to c-CSC3, probably due to intrinsic reduced expression of EGFR (Figure 5C).

### Combination of AG1478 and Crenolanib triggers apoptosis in both GBM CSC pools

**Figure 6:**
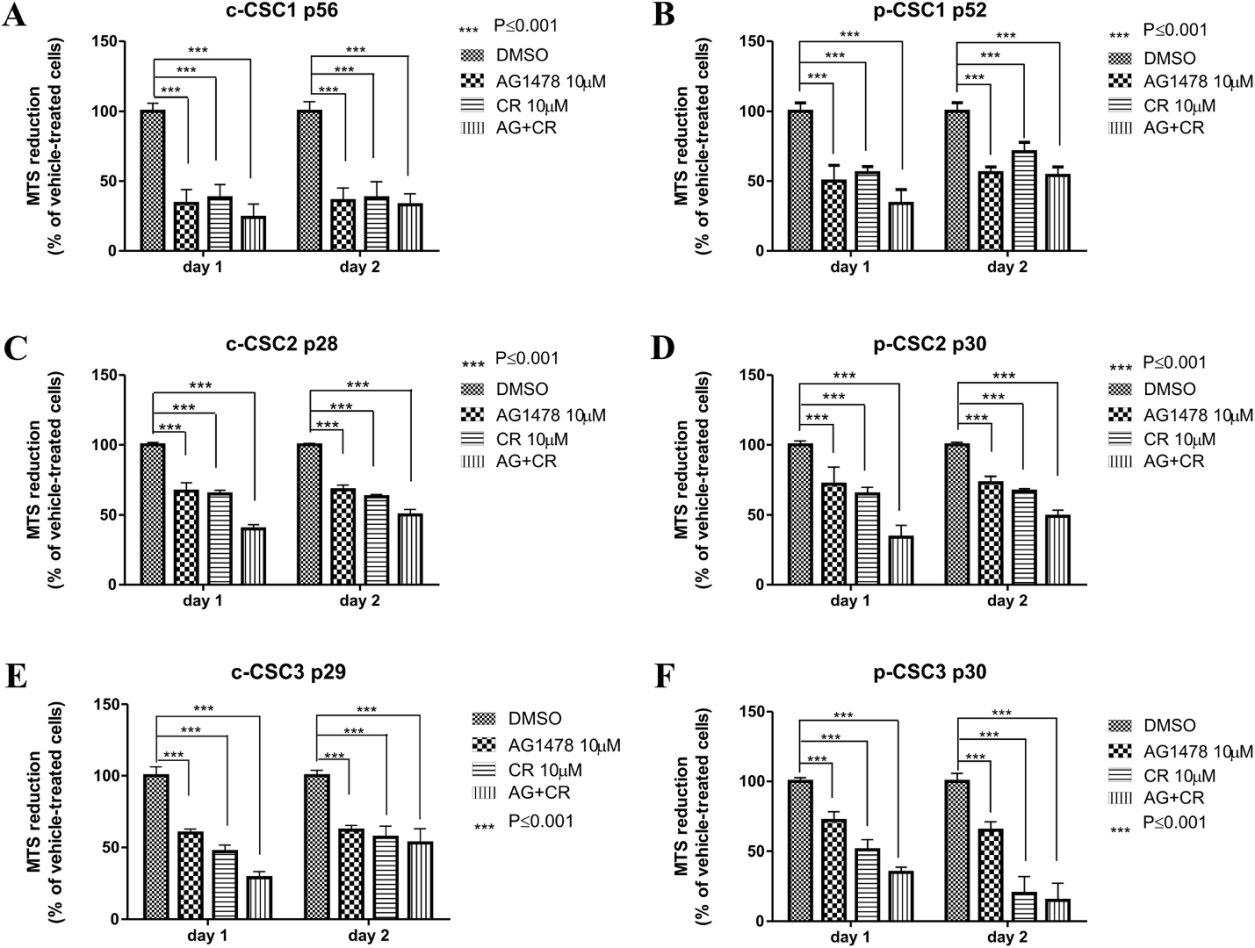
**AG1478 and Crenolanib trigger cell growth inhibition in both c-CSC and p-CSC. (A, B)** AG1478 alone or in combination with CR determine a significant antiproliferative effect in c-CSC1 and p-CSC1 either at 1 day or 2 days of treatment. No synergistic antiproliferative effect is monitored with the combination therapy. **(C, D)** AG1478 alone or in combination with CR determine a significant antiproliferative effect in c-CSC2 and p-CSC2 either at 1 day or 2 days of treatment. The combination therapy triggers a synergistic antiproliferative effect in p-CSC2 at day 1. **(E, F)** AG1478 and CR provided alone determine a significant antiproliferative effect in c-CSC3 and p-CSC3 either at 1 day or 2 days of treatment. The combination therapy triggers a synergistic antiproliferative effect in both c-CSC3 and p-CSC3 at day 1 of treatment. Error bars represent the mean ± SD (n = 3). All data shown are representative of results obtained from experiments conducted twice.

**Figure 7:**
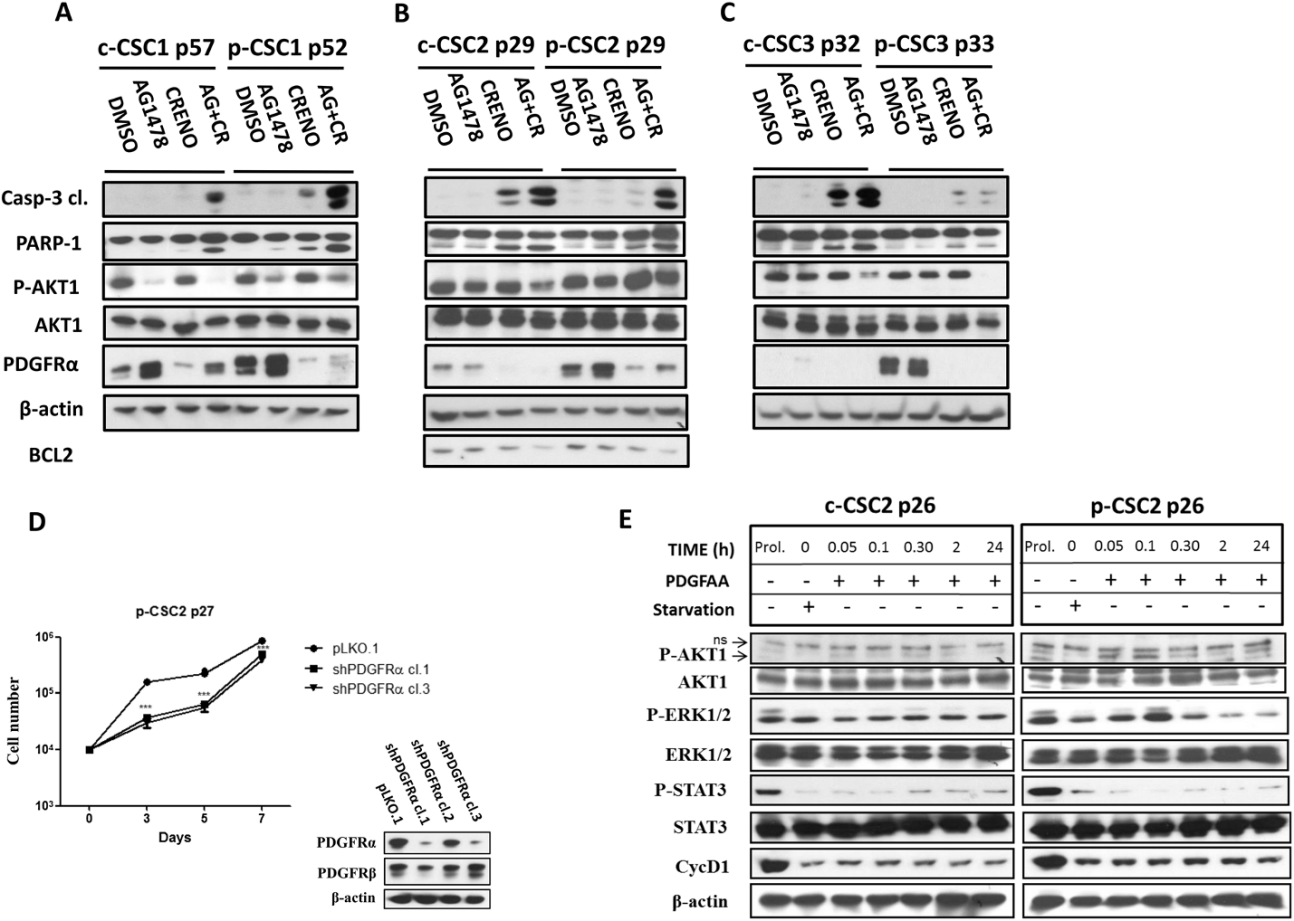
**Down modulation of PDGFR activity affects cell growth and survival of GBM CSC. (A-C)** Concurrent treatment with Crenolanib and AG1478 enhances apoptosis as monitored by Caspase-3 and PARP-1 cleavage, either in GBM c-CSC or p-CSC, except for p-CSC3. Instead, Crenolanib alone is less effective in inducing apoptosis either in c-CSC or p-CSC pools. High PDGFRα expression is a distinctive feature of p-CSC pools and its expression is de-repressed following AG1478 treatment clearly evident in case 1 and 2, while its expression is downmodulated following Crenolanib treatment in all cases reported. **(D)** Cell growth assay of shPDGFRα-p-CSC2 clones (cl.1 and 3) displays a significant reduction of cell proliferation with respect to control cells (pLKO.1). Data shown are representative of results obtained from experiments conducted two times. Western blots display downmodulation of PDGFRα in shPDGFRα-p-CSC2 clone 1 and 3, but not of PDGFRβ. **(E)** c-CSC2 and p-CSC2 starved for 2 days and undergoing PDGFAA stimulation, manifest early phosphorylation of Akt1 and Erk1/2 in p-CSC2 but not in c-CSC2. No effects were observed on STAT3 phosphorylation upon PDGFAA stimulation. Expression of Cyclin D1 protein serves as a control of cell starvation. ns denotes not specific bands.

The high PDGFRα protein expression reported in all p-CSC of the six cases examined, prompted us to test the pharmacological inhibition of PDGFRα to evaluate its impact on cell growth and survival of CSC. In this study, EGFR signaling inhibition have been shown to be more effective therapeutically than GSI-X, suggesting that the combination with Crenolanib (CR, a selective inhibitor of PDGFRα), would enhance the growth inhibitory effect especially in p-CSC, which were more resistant to treatments. As reported in Figure 6A, B, we observed a significant decrease of cell proliferation in both c-CSC1 and p-CSC1 (38 ± 9.5% and 56 ± 4.4% respectively) with CR (10 μM) alone after 1 day of treatment by MTS assay, but when used in combination with AG1478 (10 μM) the average values declined to 24 ± 9.6% and 34 ± 9.9% respectively. In addition, Western blots analysis clearly showed an induction of Caspase-3 and PARP-1 cleavage fragments, the effect of which was maximized by combining the two inhibitors (Figure 7A). Of note, is the de-repression of PDGFRα in AG1478-treated CSC1 as well as in p-CSC2 (Figure 7B), this is probably due to a compensatory activation mechanism to evade EGFR TKI. In addition, we detected a downmodulation of PDGFRα expression following Crenolanib treatment, which was a common feature in all cases examined (Figure 7A-C). We applied the same treatments to CSC2 and CSC3 (Figure 6C-F). The MTS assay monitored a significant reduction of cell proliferation to 65 ± 2.6% and 47 ± 5% in c-CSC2 and c-CSC3 respectively, while in p-CSC2 and p-CSC3 cell growth declined to 65 ± 4.8% and 51 ± 7.3% respectively with CR alone. Dual treatment was more effective than treatment alone in both CSC2 and CSC3, as reported in Figure 6C-F. Of note, as early as the first day of treatment the two agents exerted a synergistic antiproliferative effect in CSC2 and CSC3 with induction of Caspase-3 and PARP-1 cleavage and reduction of p-Akt1 in CSC3 or Bcl2 in CSC2 (Figure 7B, C).

### Modulation of PDGFRα activity regulates cell growth and survival pathways in GBM CSC

To confirm the pivotal role of PDGFRα in cell proliferation, we downregulated his expression by short hairpin RNA mediated-knockdown in p-CSC2. After a brief period of antibiotic selection, either control infect cells (pLKO.1) or two cell clones expressing shPDGFRα were plated for cell growth assessment. shPDGFRα-cells showed a significant reduction of cell growth rate compared to pLKO.1 cells, which was consistently maintained up to the seventh day in culture (Figure 7D). In order to evaluate p-CSC response to PDGFAA, c-CSC2 and p-CSC2 were undergone to growth factors (EGF and bFGF) deprivation for 2 days, and then exposed to PDGFAA (40 ng/ml) for short periods of time (Figure 7E). PDGFAA stimulation induced early activation of p-Erk1/2 and p-Akt1 in p-CSC2 within 5 minutes, which declined to basal levels later. Conversely, c-CSC2 displayed a faint response to PDGFAA.

## Discussion

In recent years, aberrant RTK expression has gained much attention in cancer stem cells biology. Several TKI and blocking antibodies anti-EGFR have been tested and others are currently in 30 ongoing clinical studies for GBM treatment, according to the website of *clinicaltrial.gov* (United States National Institutes of Health). Despite the seemingly critical role of EGFR signaling in GBM, only 10 to 20 percent of patients have shown a modest improvement by EGFR kinase inhibitors [30, 31]. These initials clinical results have demonstrated the need to understand GBM’s resistance mechanisms to EGFR inhibition as well as to other inhibitors in order to develop more effective therapies [32]. A sub-population of CD133 positive cancer stem cells with higher Notch activity has been identified in GBM [33]. Notch inhibition by γ-secretase inhibitors depleted CD133+ glioblastoma cells, making these compounds potential chemotherapeutic agents to target high-grade gliomas. A terminated phase I pharmacological study with MK-0752 (a gamma-secretase inhibitor developed by Merck), demonstrated clinical benefits in patients with advanced solid tumors, including GBM, and now combination trials are ongoing to maximize the therapeutics benefit with this novel agent [34].

PDGFRα is another critical gene in glioma biology, as it is the second most frequently mutated TRK in GBM, following EGFR. Similar to EGFR, PDGFRα has been shown to be overexpressed, amplified, mutated and rearranged in GBM. PDGFRα gene amplification could be used as a potential prognostic biomarker and therapeutic target in GBM [35]. A phase II study with a monoclonal antibody anti-PDGFRα is under investigation in recurrent GBM.

In the current study we assessed the effects of Notch1, EGFR and PDGFR inhibitors as a means of investigating GBM CSC responses to disruption of specific intracellular signaling relevant to GBM CSC maintenance. We identified, in some cases, a heterogeneous expression of NOTCH1 gene, in particular we reported sustained levels of expression in p-CSC3 and p-CSC4 with respect to relative counterparts c-CSC. In three cases deeply investigated, c-CSC pools were impaired in cell growth by GSI-X, while the relative p-CSC pools resisted treatment or delayed the inhibitory effects. The Notch1 pathway was investigated in more detail in three cases and surprisingly we found that Notch1 target Hes1 protein was not down modulated by Notch inhibition. This finding may be explained by the results of Wall et al. [28], who demonstrated that sonic hedgehog (shh)-driven stabilization of Hes1 was independent of Notch signaling and required the Shh effector Gli2. Moreover, a physiological crosstalk has been provided between Notch-Hes1 and JAK-STAT pathways in the developing central nervous system, as Hes proteins (Hes1 and Hes5) bind to STAT3 directly, thereby suggesting that Hes proteins may function as non-scaffold proteins that allow JAK2 to phosphorylate STAT3 [36]. More recently, other authors report that STAT3 and NF-κB signaling regulates the Notch pathway in glioblastoma cancer stem cells [37]. It is possible that as a consequence of this complex interactions, Notch pathway would be partially hampered by GSI-X treatment, as we detected uncertain down modulation of the cell cycle progression protein CycD1 in c-CSC2 (but not of p27), and of the anti-apoptotic protein survivin in c-CSC3 (but not of Bcl2). Based on the above results only a small fraction of cell death was monitored in GSI-treated c-CSC.

A study on 196 cases of GBM from the TCGA consortium, reports that the expression of Notch signaling components was enriched in the classical/proliferative GBM subtype characterized by EGFR+/PDGFRA- and in the proneural subtype, characterized by PDGFRA+/IDH1+ [38], indeed we did find these correlations in our GBM CSC. Accordingly to the literature, fifty percent of GBM CSC examined in the current study had shown overexpression of EGFR, which resulted independently from Notch1 activation, despite other laboratories’ reports that Notch and EGFR signaling pathways converge to regulate the same gene targets. Purow et al. [39], report that Notch1 regulates transcription of EGFR through p53 and Xu et al. [40], stated that knockdown of Notch1 expression by siRNA downregulated the expression of EGFR and the important components of its downstream pathways, including PI3K/Akt, K-Ras, Cyclin D1 and MMP9.

The high EGFR expression in GBM CSC prompted us to explore EGFR signaling as a therapeutic approach and afterwards we decided on combinatorial therapy anti-Notch and anti-EGFR. Our and other laboratories have reported that a specific inhibitor for EGFR efficiently blocked EGF-induced activation of MMP9 and reduced cancer invasiveness [40]. The inhibitory effects of AG1478 on CSC invasive ability would impact consequently also STAT3 signaling [41], as reported in the current study except for p-CSC1 and p-CSC3, for whose we did not observed any modulation of phosphorylation on Y705-STAT3 following AG1478 treatment. STAT3 signaling is also involved in GBM invasion promotive effect of IL-6 [42]. The combination of AG1478 and GSI-X exceeded the effects of monotherapy as reported in Western blots, flow cytometry and cell invasion assays. The uncertain effects mediated by Notch inhibition alone were clearly overridden by combining AG1478, which produced: i) apoptosis, ii) switch-off of p-Akt1 and p-Erk1/2 expression, and iii) reduction of CycD1 and Survivin, except in the most resistant p-CSC3 and the arrested CSC1. These effects mirrored a marked reduction of CSC invasive ability with MMP9 protein down modulation as observed in most of the cases except for p-CSC3.

EGFR has been the focus of many brain tumor studies and it is noteworthy that expression of wild-type or constitutively active mutant EGFR is rarely oncogenic as a single lesion, whereas expression of PDGFR ligands can induce tumors as a single driving event [43, 18]. PDGFs and PDGFRs deregulated expression are found even in low-grade gliomas [43–45], suggesting that this pathway is possibly an early oncogenic event, in contrast to EGFR which is much more commonly found in high-grade gliomas [46]. Here we report for the first time the inherent overexpression of PDGFRα in GBM p-CSC with respect to c-CSC in all six patient samples examined, suggesting the dominance of its signal in proliferation and maintenance of GBM and in particular of peritumor tissue-derived CSC. Recently, it has been reported that tyrosine-protein phosphatase non-receptor type 11 (SHP-2) and PI3K mediate PDGFRα-promoted glioma tumor growth and invasion [47]. Based on our results and those of other authors, we hypothesize that the combinations of abnormal expression and activation of growth factor receptors such as: EGFR, PDGFRα, PDGFRβ and Notch1 in GBM CSC or -derived cell lines may influence cell response to targeted therapies, thereby limiting the efficacy of single anti-EGFR or anti-PDGFR or anti-Notch1 therapies as we have seen in this study [48, 49].

To demonstrate that PDGFRα was relevant to GBM CSC maintenance, we tested a potent PDGFRα signaling inhibitor Crenolanib in the most studied cases (cases 1-3). A significant reduction of cell growth of either c-CSC or p-CSC was reported, with remarkable effects on the 2^nd^ day of treatment on p-CSC3. The combination therapy with AG1478 enhanced apoptosis induced by CR alone, supporting the hypothesis that targeting more RTKs would probably weaken the tumor growth. It is important to note the up regulation of PDGFRα protein observed in some cases in response to EGFR inhibition, which suggests its role in mediating AG1478 resistance in GBM. Recently, it has been reported that this event is emerging as a frequent, non-genetic mechanism of targeted cancer drug resistance, as reported in GBM CSC, colorectal cancer cells and in non-small cell lung cancer [23]. In addition, genetic knockdown of PDGFRα in p-CSC2 caused slowdown of cell growth rate and exogenous PDGFAA stimulation of starved GBM CSC promoted p-Erk1/2 and p-Akt1 activation in p-CSC, confirming the concept that PDGFRα activation provided survival signals in p-CSC *vs*. c-CSC.

## Conclusions

Our results collectively support a key role of EGFR and PDGFRα signaling in survival of Glioblastoma cancer stem cells. PDGFRα was overexpressed in GBM peritumor tissues derived-CSC compared to counterparts GBM c-CSC, indicating this pathway a pivotal therapeutic target in GBM. We also provide the rationale for simultaneous targeting of EGFR and PDGFR, with prospective of an improvement of survival and quality of life of GBM patients.

## Material and methods

### Ethical statement

Procedures for collection of adult human GBM CSC were approved by the Ethical Committee of the Catholic University of Rome as reported previously [24]. Informed consent was obtained and all patients were fully aware of the aims and scope of this work. The ethical principles of the declaration of Helsinki were strictly followed.

### Cell culture and treatment of human Glioblastoma cancer stem cells

We have used the same clinical materials reported in our previous papers [24, 25]. In brief, the CSC cells were retrieved from adult patients affected by GBM and undergoing craniotomy at the Institute of Neurosurgery, Catholic University-School of Medicine of Rome, Italy. Dissociated cells were cultured in the presence of human recombinant EGF (20 ng/ml; PeproTech, Rocky Hill, NJ), human recombinant bFGF (10 ng/ml; PeproTech), in DMEM/F12 (1:1) serum-free medium (Invitrogen, Carlsband, CA) containing L glutamine 2 mM, glucose 0.6%, putrescine 9.6 ug/ml, progesterone 0.025 mg/ml, sodium selenite 5.2 ng/ml, insulin 0.025 mg/ml, apo-transferrin sodium salt 0.1 mg/ml, sodium bicarbonate 3 mM, Hepes 5 mM, BSA 4 mg/ml, heparin 4 ug/ml (all purchased by Sigma-Aldrich). Floating neurospheres were dissociated with Accutase at 37°C (Merck-Millipore). In some cases, neurospheres were passaged up to passage P60 and the experiments were performed between P14 and P60. Cell starvation was planned for 2 days in Stem Medium w/o EGF and bFGF. Subsequently, PDGF-AA was added (40 ng/ml, Peprotech) for different time points (5′, 10′, 30′, 120′ and 24 hours). Cells treatments were performed with GSI-X (also named L-685,458, Calbiochem), AG1478 (Calbiochem) and Crenolanib (CP-8685596, Selleckchem).

### ShRNA, transfection and lentivirus production

The experiments on RNA interference were performed using Mission Lentivirus-based shRNA for PDGFRα (NM_006206-Sigma-Adrich). We amplified three DNA clones (clones numbers: TRCN0000195132, TRCN0000196272, TRCN0000196928) and pLKO.1-puro, as a control for infection. We selected puromycin resistant p-CSC2 expressing three different shRNA sequences, but only two were able to donwmodulate PDGFRα (TRCN0000195132 and TRCN0000196928). Human embryonic kidney (HEK)-293 T cells in log-phase growth were transiently transfected using standard LipofectAmine reagent (Invitrogen), with lentivirus-based shRNA (and with pLKO.1 as control) plus helper plasmids (Invitrogen Packging Plasmids, Carlsbard, CA). Media containing the virions were harvested two days after cell transfection and transferred directly onto p-CSC2. Lentiviral infection was performed in the presence of polybrene solution at 8 μg/ml (Sigma-Aldrich) and the antibiotic puromycin (Euroclone) was added to the cells at 1 μg/ml for a week to select CSC expressing shRNA sequences.

### Western blots

GBM CSC seeded as single cells (1 × 10^6^/dish) were left for 1 day in proliferation medium before treating them for 1-2 days with the inhibitors, either singly or in combination. Afterwards, cells were collected and washed with PBS plus proteases inhibitors before protein extraction in 100-200 μl of lysis buffer (1% NP-40, 0.01% SDS, 20 mM Tris–HCl pH 7.4, 300 mM NaCl, 1 mM EDTA, 1 mM Na_3_VO_4_ and protease inhibitors cocktail - from Sigma–Aldrich). Then, cells were sonicated with two pulses of 5 sec at 50% of amplitude (Sonics and Materials, Newtown, CT). Equal amounts (30 μg/lane) of total protein extracts, determined by Bio-Rad protein Assay (Bio-Rad, Munchen, Germany), were loaded on NuPAGEBis-Tris gels (Invitrogen), and transferred on Hybond-P Extra membrane (Amersham Biosciences, GE Healthcare Life Science-Buckinghamshire, UK). Filters were immunoblotted using the following primary antibodies: rabbit anti-NICD1, rabbit anti-HES1, rabbit anti-DLL1, rabbit anti-EGFR, rabbit anti-pY1068-EGFR, rabbit anti-PDGFRα, rabbit anti-PDGFRβ, rabbit anti-T202/Y204-ERK1/2 and anti-ERK1/2, rabbit anti-Y705-STAT3 and rabbit anti-STAT3 and anti-Caspase3, (all purchased from Cell Signaling, MA-USA), mouse anti-S473-AKT1 and rabbit anti-AKT1 (Calbiochem), rabbit anti-BCL2 (Millipore), rabbit anti-survivin and anti-MMP9 (Abcam), rabbit anti-Cyclin D1 and anti-p27 (Santa Cruz-USA), mouse anti-β-actin and anti-GAPDH (SIGMA). After three washing with TBS-Tween buffer, immuno-reactive proteins were detected using rabbit anti-mouse, donkey-anti-rabbit and donkey anti-goat horseradish peroxidase-conjugated secondary antibodies directed to the appropriate primary antibodies (Jackson Immunoresearch Laboratories, West Grove, PA). The proteins were then visualized using the chemiluminescence system (Millipore). Gels and Images acquisition was done by HP Photosmart Essential Ver. 1.12 and Adobe Photoshop CS5 respectively. The densitometric analysis of protein bands normalized against to β-actin protein levels were performed from three independent experiments using the ImageJ software (NIH, USA).

### Cell proliferation assays

For cell proliferation assay, neurospheres were dissociated into single cells and 1 × 10^4^ cells/well were plated in triplicate on 60 mm plates. Cells were harvested and counted at different time points (3, 5, 7 days in vitro) in growth medium, considering the starting time as the day after plating. For pharmacological studies we used the CellTiter 96 Aqueous One Solution Reagent (Promega), a cell proliferation colorimetric assay containing a novel tetrazolium compound MTS. Briefly, 2 × 10^4^ cells/well were plated in triplicate for each group on 12-well plates and the drugs or DMSO-vehicle were added the day after plating. Before harvesting cells, they were incubated with 100 μl/ml of MTS at 37°C for approximately 1 hour. The metabolically active cells reduced MTS into a soluble formazan product, whose absorbance was measured at 490 nm in a plate reader (Bio-Rad). These experiments were performed three times and each time in triplicate.The absorbance values of the collected samples were subtracted from the background absorbance of medium-only control and expressed as % of control and calculated as mean average ± SD (n = 3).

### RT-PCR and custom RT-PCR array

Total RNA was extracted using Triazol and by RNeasy mini kit (Qiagen, USA). cDNAs were obtained using QuantiTect Reverse Transcription kit (Qiagen, USA). Real time reverse transcriptase PCR (RT-PCR) was carried out in triplicate using SYBR Hi-ROX kit (Bioline, UK). RT-PCR and a custom RT-PCR array (Microfluidic Card, Applied Biosystems, CA) were performed with a 7900HT instrument equipped with SDS2.2 software (Applied Biosystems, CA). The PCR primers used for RT-PCR were the following: (Forward) hTBP: GAACATCATGGATCAGAACAACA, (Reverse) hTBP: ATAGGGATTCCGGGAGTCAT; (Forward) hGAPDHAGCCACATCGCTCAGACA; (Reverse) hGAPDH: GCCCAATACGACCAAATCC, as internal controls. (Forward) hEGFRvIII: GGCTCTGGAGGAAAAGAAAG*GTAAT*; (Reverse) hEGFRvIII: TCCTCCATCTCATAGCTGTCG; (Forward) hEGFR: GCCTTGACTGAGGAACAGCA; (Reverse) hEGFR: TTTGGGAACGGACTGGTTTA; (Forward) hNOTCH1: CGCACAAGGTGTCTTCCAG; (Reverse) hNOTCH1: CGGCGTGTGAGTTGATGA. Relative levels of expression were obtained by normalization with respect to selected housekeeper genes (GAPDH, TBP) by using the ∆Ct method following manufacturer’s guide.

### Cell invasion assay

For an invasion assay, 1 × 10^5^ cells were resuspended in 0.3 ml of Stem Medium with inhibitors or DMSO-vehicle and placed in triplicate into the top chamber of matrigel-coated transwell insert (Millipore). The bottom wells contained 0.4 ml of Stem Medium with EGF and bFGF, or without these media as controls for the experiment. After 72 h, cells on the top surface of the filter were removed with a cottonswab. Thereafter, the filters were fixed and stained with crystal violet and subsequently washed to collect the staining solution. OD values, proportional to the number of cells, were measured on a plate reader with a 550 nm filter (Bio-Rad). These experiments were performed three times and each time in triplicate. The absorbance values were calculated as mean ± SD (n = 3). These experiments were repeated three times and each time in triplicate.

### FACS analysis

Cells were fixed with 0.5 ml of cold Methanol/Acetone Solution (1:5) and left at 4°C for at least 1 hour, then centrifuged at 950 RPM for 5 minutes. The cell pellet was resuspended in 100 μl of PBS, 375 ul of Rnase A (100 μg/ml) and 25 μl of Propidium Iodide (1 mg/ml). Cells were incubated at room temperature for 15 minutes and left in the dark at 4°C until FACS analysis. Samples were acquired on FacsCalibur (BD Becton Dickinson) at 488 nm. The acquired FACS data were analyzed by ModFit LT software (Verity Software House, Inc.) to determine the percentage of cells sub G1, G1, S and G2/M phases. These experiments were performed twice for each sample.

### Statistical analysis

Statistical analysis was performed with Prism5 (GraphPad) and Microsoft Office Excel 2007. All data shown are representative of results obtained from experiments conducted two or three times as specified in the specific sections. The results were analyzed by either One-way Anova and Newman-Keuls post-tests or Two-way ANOVA and Bonferroni’s post tests. Data are expressed as mean ± standard deviation (SD) and P values ≤0.05 (*), ≤0.01 (**), ≤0.001 (***) were considered statistically significant. To evaluate the synergistic effect of the combination of two drugs on cell growth we referred to Lama et al. [50].

## Electronic supplementary material

Additional file 1: Figure S1: GSI-X did not modulate survival signals and cell growth of p-CSC3. (A) Increasing concentrations of GSI-X did not affect Erk1/2 and Akt1 phosphorylation. (B) Cell growth of p-CSC3 was not affected by high doses of GSI-X. Values are expressed as mean of ± SD (n = 4). ns denotes not specific bands. (TIFF 2 MB)

## Abbreviations

GBM: Glioblastoma multiforme
c-CSC: core cancer stem cells
p-CSC: peritumor cancer stem cells
GSI-X: Gamma secretase inhibitor-X
NICD1: Notch intracellular domain 1
HES1: Hairy enhancer of split 1
DLL1: Delta like ligand 1
PSEN1: Presenilin 1
RTK: Receptor tyrosine kinase
EGFR: Epidermal growth factor receptor
PDGFR: Platelet derived growth factor receptor
TKI: Trosine kinase inhibitor
CR: Crenolanib.
